# Risk factors and clinical significance of refractory pain in patients with bone metastases: a comprehensive meta-analysis

**DOI:** 10.3389/fneur.2025.1517279

**Published:** 2025-04-24

**Authors:** Qiju Li, Qingqing Liu, Liu Yang, Qin Li, Aimin Zhang

**Affiliations:** ^1^Chengdu Xinhua Hospital Affiliated to North Sichuan Medical College, Chengdu, China; ^2^Department of Anesthesiology, Sichuan Clinical Research Center for Cancer, Sichuan Cancer Hospital & Institute, Sichuan Cancer Center, University of Electronic Science and Technology of China, Chengdu, China

**Keywords:** refractory cancer pain, bone metastases, risk factors, meta-analysis, incidence, pain management

## Abstract

**Background:**

Refractory cancer pain, especially bone pain, presents a major clinical challenge that is difficult to manage despite the use of multimodal analgesic strategies. This meta-analysis aims to estimate the prevalence of refractory cancer pain in this patient population and to identify potential predictors that may increase the likelihood of developing such pain. In addition, we performed a systematic review of previous studies that delve into more effective pain strategies.

**Methods:**

This meta-analysis and systematic review were conducted in accordance with the PRISMA guidelines. A comprehensive search was performed using PubMed, Web of Science, Embase, and the Cochrane Library on risk factors for refractory metastatic bone pain. The inclusion criteria focused on studies reporting the incidence and/or risk factors associated with refractory cancer pain, providing relevant statistical measures such as odds ratios (OR), hazard ratios (HR), or relative risks (RR). The methodological quality of the studies was assessed using the Newcastle–Ottawa Scale (NOS), and a random-effects meta-analysis was conducted using the R programming language.

**Results:**

The present study included eight studies with a cumulative sample size of 2,774 patients. The aggregated incidence of refractory cancer pain was found to be 70% [95% confidence interval (CI): 42 to 88%] using a random-effects model, highlighting a significant prevalence of pain that remains unresponsive to treatment. Notably, the heterogeneity among the included studies was considerable (*I*^2^ = 98%, *τ*^2^ = 2.7198). The analysis also identified several critical predictors of refractory cancer pain. The presence of multiple bone metastases was consistently linked to an increased likelihood of refractory cancer pain with an OR of 3.94 (95% CI: 2.64–5.87). Similarly, lytic bone metastases demonstrated a high OR of 5.99 (95% CI: 3.17–11.30). Furthermore, there was a strong correlation between the occurrence of refractory cancer pain with severe acute pain (OR = 219.20, 95% CI: 0.26–188127.63), breakthrough pain (OR = 16.44, 95% CI: 0.60–448.07), and psychological comorbidities such as depression (OR = 3.91, 95% CI: 1.22–2048.64) and anxiety (OR = 4.22, 95% CI: 1.22–2048.64).

**Conclusion:**

Refractory cancer pain, observed in approximately 70% of patients with bone metastases, poses a significant clinical challenge. Refractory cancer pain predictors include the presence of multiple and lytic bone metastases, severe acute pain, breakthrough pain, and psychological comorbidities. Collectively, our findings highlight the need for improved pain management strategies that address both the physical and psychological aspects of cancer pain.

## Introduction

Cancer metastasizes to the bone in more than a third of all cancer patients, with a powerful strong association with breast, prostate, and lung cancers. Bone metastases are a common and severe complication of advanced-stage cancers, significantly affecting patient morbidity and quality of life (1, 2). Osseous metastases contribute significantly to patient morbidity, manifesting as pathological fractures, hypercalcemia, spinal cord compression, and pain (3, 4). These complications can profoundly impact patients’ quality of life and functional status, particularly regarding skeletal-related events (SREs) such as musculoskeletal pain and spinal cord compression (5, 6). Managing associated pain often poses a challenge due to its chronic nature and resistance to conventional therapeutic interventions (7). Chronic pain can substantially impair physical, emotional, and social functioning, underscoring the necessity for effective therapeutic interventions (8).

The pathophysiology of pain associated with bone metastasis stems from a complex interplay that involves interactions among bone tissues, neural structures, vasculature, and neoplastic cells (9, 10). Metastatic tumor cells within bone tissue stimulate osteoclast activity, developing osteolytic lesions. This process contributes to bone degradation, structural instability, and increased fracture risks, all of which could contribute significantly to pain (11). Conversely, some cancer types can cause osteoblastic metastases, characterized by increased bone formation activity. This process could result in sclerosis and heightened pain due to nerve compression (12). Moreover, inflammation caused by cancer, tumor-induced growth pressure, and nerve infiltration are critical factors in the pain process (13). Patients with bone metastases may consequently experience continuous pain, breakthrough pain, and incident pain, necessitating intensive and combination analgesic therapy (14).

Refractory cancer pain remains an unresolved clinical problem (15). While opioids, bisphosphonates, and radiotherapy are the primary treatments for cancer pain management (16), they often fail to adequately control pain for a significant number of patients (17, 18). This not only diminishes patients’ quality of life but also carries prognostic implications, as those suffering from more severe refractory cancer pain often have a lower survival rate (19). In clinical practice, understanding the factors that influence the onset, severity, and duration of pain in patients with bone metastases is essential for developing effective pain control and improved patient outcomes (20).

Nonetheless, while the importance of addressing refractory cancer pain in patients with bone metastases is recognized in clinical practice, the pain predictors remain insufficiently discussed (21). Several attempts have been made to examine the factors that may influence the degree and duration of pain in patients with bone metastases. These include the extent of bone involvement, patient characteristics, cancer stage, previous therapies, and comorbidities (22). However, such data remain inconclusive, highlighting the need for a meta-analysis of data from multiple studies to identify the most credible predictors of refractory cancer pain and their potential clinical relevance.

The absence of well-defined guidelines for assessing patients at higher risk for refractory cancer pain results in suboptimal pain management practices. Clinicians face significant challenges when standard pain management medications and supportive measures fail to effectively control pain in patients. To address this gap and provide comprehensive insights into the data and trends related to predictors of refractory metastatic bone pain, we performed a systematic review and meta-analysis. Identifying these predictors will enable clinicians to assess patient risk better, implement more targeted pain management interventions, and potentially improve quality of life and survival rates.

## Aims and objectives

The main objective of this systematic review and meta-analysis is twofold: to identify potential predictors of refractory cancer pain in patients with bone metastases and to assess the clinical applicability of these findings. Specifically, the research aims to:

(1) Identify the key risk factors for developing refractory pain in patients with bone metastases.
(2) Quantify the association between these risk factors and refractory pain incidence.
(3) Evaluate the impact of refractory cancer pain on clinical outcomes, including quality of life and overall survival.
(4) Provide recommendations for optimizing pain management strategies in patients with bone metastases.
Synthesize evidence on how pain predictors can guide clinical decision-making in cancer care.

## Methodology

The article adhered rigorously to the Preferred Reporting Items for Systematic Reviews and Meta-Analyses (PRISMA) guidelines.

### Search strategy

We thoroughly searched multiple databases to find studies on the causes of refractory cancer pain in bone metastases. The electronic databases searched included PubMed, Embase, the Cochrane Library, and Web of Science. The search utilized both keywords and MeSH terms such as “refractory cancer pain,” “bone metastases,” “pain predictors,” “risk factors,” “meta-analysis” and “systematic review.” The Boolean connectors AND and OR were applied to the keywords to enhance the effectiveness of the search strategy.

The articles included in the study were not restricted on the year of publication; however, only articles published in English were considered. Additionally, the reference lists of the identified studies were screened during the database search to identify potentially overlooked articles.

### Inclusion and exclusion criteria

To ensure the relevance and quality of the included studies, the following inclusion and exclusion criteria were used:

#### Inclusion criteria

(1) Studies evaluating the predictors or risk factors for refractory pain in patients with bone metastases.
(2) Studies provide relevant statistical measures, such as odds ratios (OR), hazard ratios (HR), or relative risks (RR).
(3) Randomized controlled trials (RCTs), cohort studies, case–control studies, or large observational studies.
(4) Studies conducted on human populations.
(5) Articles published in English.

#### Exclusion criteria

(1) Studies with small sample sizes.
(2) Reviews, commentaries, editorials, or case reports.
(3) Studies with inadequate reports on statistical measures.
(4) Animal studies or preclinical research.

### Study screening

All records retrieved from the initial database search were exported into the reference management software EndNote. Duplicates were removed, and the remaining articles were further sifted based on their titles and abstracts to determine study eligibility. This screening process was conducted under the supervision of two independent reviewers, with a third reviewer assisting in cases of disagreement.

After the initial screening based on titles and abstracts, the full texts of the potential studies were obtained and evaluated for inclusion. The final review and meta-analysis included only those studies that met all the inclusion criteria.

### Data extraction

Data extraction was carried out independently by two reviewers using a predesigned data extraction sheet. The following information was extracted from each study:

(1) Study characteristics: author(s), year of publication, study design, sample size, country/region.
(2) Patient demographics: age, sex, cancer type, disease stage.
(3) Pain-related outcomes: refractory cancer pain incidence, pain assessment tools used, definition of refractory pain.
(4) Risk factors or pain predictors: biological, clinical, and treatment-related factors.
(5) Statistical measures: odds ratios (OR), hazard ratios (HR), confidence intervals (CI), and *p*-values.
(6) Duration of follow-up and timing of outcome assessment.
(7) Information on adjustments for confounders and whether multivariable analyses were conducted.

Both reviewers cross-checked the accuracy of all extracted data, and any discrepancies were resolved through discussion.

### Quality assessment using the Newcastle–Ottawa Scale

The risk of bias in the included cohort and case-control studies was specifically evaluated by the Newcastle–Ottawa Scale (NOS). The NOS assesses the quality of studies in three main areas:

(1) Selection: This includes case/control definition, representativeness of the exposed cohort, and selection of non-exposed controls.
(2) Comparability: Proposals for comparing cases and controls in terms of design or analysis.
(3) Outcome: Assessment of the outcome, follow-up period and adequacy of cohort follow-up.

These benchmarks were applied to each study, with each receiving a score out of 9 points. Studies were categorized as high quality if they scored 7 or higher, moderate quality if they scored between 5 and 6, and low quality if they scored below 5.

### Data synthesis

The extracted data were analyzed using statistical analysis using R software. A meta-analysis was conducted to combine the effect sizes of the risk factors across the studies. For each risk factor, OR, HR, or their 95% CI were extracted. Meta-regression was then performed using a random-effects model to address heterogeneity among the studies. Inter-study variability was assessed using *I*^2^ statistic, which measures the proportion of variation between studies not attributable to pure chance. An *I*^2^ value greater than 50% was deemed significant enough to indicate heterogeneity. In cases of high heterogeneity, a sensitivity analysis was conducted to identify the potential causes. The presence of publication bias was evaluated using a funnel plot and Egger’s test. If asymmetry was observed in the funnel plot, further investigation was conducted to determine whether the results were due to publication bias or other factors.

## Results

### Study characteristics

**Figure 1:**
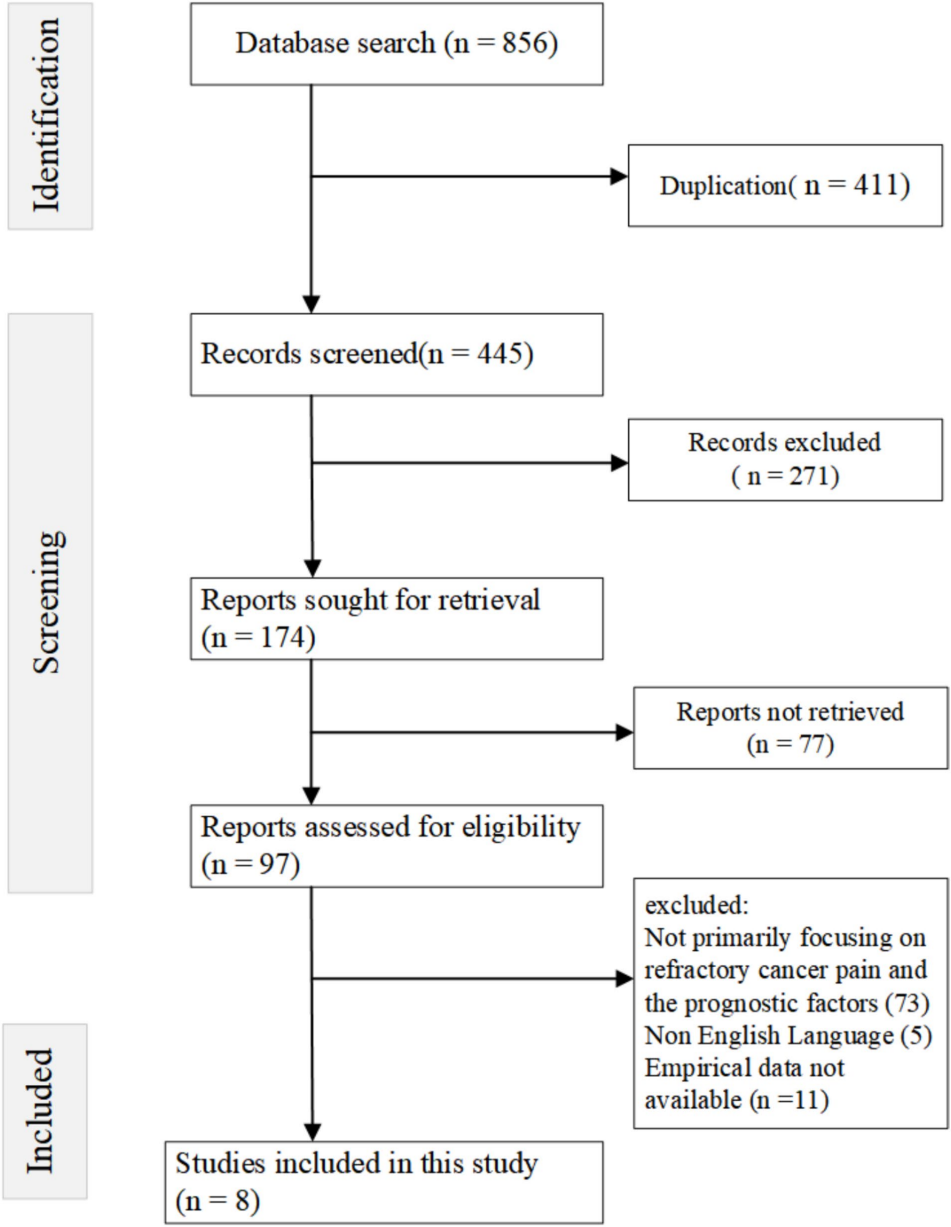
Flowchart of the literature retrieval process.

A comprehensive search across various databases yielded a total of 856 studies. After applying the inclusion and exclusion criteria, eight studies comprising of prospective observational studies, retrospective studies, pooled analyses, and cohorts were included in this meta-analysis. These studies investigated a range of antecedents and risk factors associated with refractory cancer pain in cancer patients with bone metastases. All studies reviewed in this paper were published between 1996 and 2019, with locations including the USA, China, Japan, Taiwan, and Italy. The population sample sizes varied, with the smallest study involving 31 patients (23) and the largest involving 1,445 patients (24). These studies focused on the antecedents of pain and skeletal complications, pain relief interventions, survival, and the effects of individual clinical interventions in patients with bone metastases. Various cancer types were represented, including prostate, lung, breast, and head and neck cancers; however, particular emphasis was placed on managing refractory cancer pain and skeletal events in patients with advanced or metastatic malignancies (as shown in Table 1 and Figure 1).

**Table 1:** Quality assessment of included studies using NOS.

Study	Selection	Comparability	Outcome	Total score (out of 9)	Quality
Shi et al. (30)	★★★☆	★☆	★★★	7/9	High
Berruti et al. (26)	★★★☆	★★	★★☆	8/9	High
Habib et al. (28)	★★★☆	★☆	★★★	7/9	High
Cramer et al. (29)	★★★☆	★★	★★★	8/9	High
Akakura et al. (25)	★★☆☆	★☆	★☆☆	5/9	Moderate
Tsai et al. (23)	★★☆☆	★☆	★★☆	6/9	Moderate
Parkes et al. (24)	★★★☆	★★	★★★	8/9	High

### Patient demographics and cancer types

The included studies primarily involved middle-aged to older patients, ranging from an average of 58 to 73 years. Most articles analyzed focused on male participants (25–27) while research on breast cancer included female participants (24, 28). Lung, breast, and prostate cancers were the main cancers of interest, especially among metastatic patients. Most of these patients had bone metastases affecting both the axial and appendicular regions. Moreover, studies by Parkes et al. (24) and Cramer et al. (29) examined the various skeletal involvements and their effects on survival and pain control. Notably, it was found that 71% of the breast cancer patients identified in the study by Parkes et al. (24) reported pain symptoms at the time of their bone metastasis diagnosis.

### Refractory cancer pain predictors and skeletal complications

As depicted in Table 2, these studies included in the meta-analysis consistently identified several significant predictors of refractory cancer pain in patients with bone metastases. These predictors include the severity of bone lesions, the nature of the metastasis, the initial level of pain experienced by the patient, and the patient’s performance status. For instance, in patients with prostate cancer, bone pain and the extent of bone involvement were identified as key predictors of SREs, as confirmed by Berruti et al. (26) who found that higher pain scores and greater bone involvement correlated with increased incidence of SREs (HR = 1.13, *p* < 0.0001, HR = 1.16, *p* < 0.0001).

**Table 2:** Study characteristics of studies included in the present study.

Study title	Study	Year of Publication	Study design	Country/region	Sample size	Mean age (range)	Sex (male/female %)	Cancer type	Disease stage	Previous treatment	Comorbidities	Incidence of refractory pain	Definition of refractory pain	Pain assessment tool	Risk factors identified	Statistical measures (OR, RR, HR, CI, *p*-values)	Duration of follow-up	Timing of outcome assessment	Adjustment for confounders	Multivariable analysis
Characteristics and prognostic factors for pain management in 152 patients with lung cancer	Shi et al. (30)	2016	Retrospective study	China	152	Median age: 58 (range: 32–81)	65.1% male, 34.9% female	Non-small-cell lung cancer (86.8%), small-cell lung cancer (13.2%)	Stage IV: 73.7%, stage I-III: 17.1%	Opioids, NSAIDs	Not specified	18.4%	Numeric Rating Scale ≤3 or <3 breakthrough doses/day	Numeric Rating Scale (0–10)	Bone metastases, severe pain, breakthrough pain	OR: bone metastases (OR = 3.58, *p* = 0.029), severe pain (OR = 3.88, *p* = 0.005)	3 days of analgesic treatment	3 days post-treatment	Yes	Yes
Pain predicts overall survival in men with metastatic castration-refractory prostate cancer	Halabi et al. (27)	2008	Pooled analysis of 3 phase III RCTs	USA	599	Median age: 71 (range: 64–75)	Not reported	Metastatic castration-refractory prostate cancer	Advanced with bone metastases (90%)	Opioids, mitoxantrone, hydrocortisone	Not specified	38%	Pain interference scores ≥17 on Brief Pain Inventory	Wisconsin Brief Pain Inventory (BPI)	Bone metastases, high pain interference, opioid use, poor performance status	HR for death with high pain: 1.43 (95% CI: 1.17–1.74, *p* = 0.001)	Median survival: 17.6 months vs. 10.2 months	Baseline and follow-up	Yes	Yes
Predictive factors for skeletal complications in hormone-refractory prostate cancer patients with metastatic bone disease	Berruti et al. (26)	2005	Prospective observational study	Italy	200	Median age: 73 (range: 52–92)	100% male	Hormone-refractory prostate cancer with bone metastases	Advanced metastatic disease	LHRH-A, antiandrogens, chemotherapy, steroids	Not specified	88.5% with bone pain	Not specified	Pain questionnaire (0–19 scale)	Bone pain, extent of bone metastases, serum alkaline phosphatase	HR for skeletal-related events: pain score (HR = 1.13, *p* = 0.000), bone metastases (HR = 1.16, *p* = 0.000)	Median 7 months	Every 3 months	Yes	Yes
Risk factors for severe acute pain and persistent pain after surgery for breast cancer	Habib et al. (28)	2019	Prospective observational study	USA	124	Mean: 59 years	100% female	Breast cancer (surgery)	Not applicable	Preoperative radiotherapy (6%), chemotherapy (28%)	Not specified	57.26%	Pain score ≥ 3 or impact on daily life ≥6 months	Numeric Rating Scale (0–10)	Severe acute pain, chemotherapy, radiation, surgery duration	OR for persistent pain: acute pain (OR = 5.39, *p* = 0.001), radiation (OR = 3.39, *p* = 0.023)	1 year	1, 3, 6, 12 months	Yes	Yes
Pain in head and neck cancer survivors: prevalence, predictors, and quality-of-life impact	Cramer et al. (29)	2018	Cohort study	USA	175	Median age: 65 years	70.9% male, 29.1% female	Head and neck cancer	Stage III-IV: 67.4%	Surgery, radiation, chemoradiation	Depression, anxiety	45.1, 11.5% with severe pain	Pain requiring narcotics or not controlled	UW-QOL	Trimodality treatment, major depression, anxiety, poor recreation	OR: trimodality treatment (OR = 3.55, *p* < 0.05), depression (OR = 3.91, *p* < 0.01), anxiety (OR = 4.22, *p* < 0.01)	Median 6.6 years post-diagnosis	During clinic appointments	Yes	Yes
Pain caused by bone metastasis in endocrine-therapy-refractory prostate cancer	Akakura et al. (25)	1996	Retrospective study	Japan	48	Mean age: 71.5	100% male	Endocrine-therapy-refractory prostate cancer	Advanced metastatic disease	Surgical or medical castration, diethylstilbestrol	Not specified	100%	WHO three-step analgesic ladder	WHO three-step approach	Time to requirement for analgesics, tumor marker doubling time, alkaline phosphatase	HR for shorter survival: *p* < 0.01	Followed until death	Time to need for analgesics	Yes	Yes
Prognostic and predictive factors for clinical and radiographic responses in patients with painful bone metastasis treated with magnetic resonance-guided focused ultrasound surgery	Tsai et al. (23)	2019	Retrospective study	Taiwan	31	Median age: 60 years	58.1% male, 41.9% female	Bone metastases (various primary cancers)	Advanced metastatic disease	Chemotherapy, hormonal therapy	Not specified	83.9% clinical response, 67.7% radiographic	Pain score ≥4 on NRS	Numerical Rating Scale (NRS)	Pretreatment Karnofsky performance status (KPS), lesion coverage volume factor (CVF)	OR for clinical: KPS (OR = 1.220, *p* = 0.019), radiographic: CVF (OR = 1.183, *p* = 0.006)	3 months post-treatment	1, 2 weeks; 1, 2, 3 months	Yes	Yes
Prognostic factors in patients with metastatic breast cancer with bone-only metastases	Parkes et al. (24)	2018	Retrospective cohort study	USA	1,445	Median age at diagnosis: 49.3 years	99% female, 1% male	Breast cancer with bone-only metastases	Advanced metastatic disease	Bisphosphonates, denosumab, pamidronate	Not specified	71%	Pain medications or clinical notation of pain	Not specified	Multiple bone metastases, lytic bone metastases, metastases in both axial and appendicular skeleton	OR: lytic metastases (OR = 1.79, *p* = 0.001), multiple metastases (OR = 1.37, *p* = 0.03), HR for death: multiple (HR = 1.78, *p* < 0.0001)	Median 6 months minimum	Baseline and follow-up visits	Yes	Yes

Meanwhile, Parkes et al. (24) found that patients with lytic bone metastases had 1.79 times greater odds of experiencing pain compared to those with blastic or sclerotic metastases (95% CI: 1.31–2.43, *p* = 0.001). In addition, multiple bone metastases were critical in assessing pain levels, given that patients with multiple metastases were more likely to experience pain than those with a single metastasis (OR = 1.37, *p* = 0.03). Similarly, Tsai et al. (23) identified lesion coverage volume factor as an independent predictor of radiographic response (OR = 1.183, *p* = 0.006), highlighting the importance of tumor load in determining disease outcomes.

### Impact of treatment modalities on pain and survival

Pain management regimens varied across the studies, with different treatment options being trialed. For instance, Habib et al. (28) examined the effects of chemotherapeutic treatment, radiotherapy, and surgical intervention on acute pain and persistent pain in breast cancer patients, finding that patients with severe acute pain were 5.4 times more likely to develop persistent pain (OR = 5.39, *p* = 0.001). Patients who underwent post-discharge radiation therapy exhibited a 3.4-fold increase in the likelihood of experiencing persistent pain (OR = 3.39, *p* = 0.023), confirming the impact of intensive treatments on pain endurance. Halabi et al. (27) studied the effects of opioid medications and pain interference on the survival of patients with metastatic castration-refractory prostate cancer. They found that patients with high pain interference scores faced a 43% increased risk of death, *p* = 0.001, HR = 1.43. Similarly, Akakura et al. (25) reported that the time for analgesic requirement was an independent predictor of shorter survival, highlighting the importance of effective pain management as a prognostic factor in patients with bone metastases.

### Survival outcomes and prognostic factors

Mortality was studied comprehensively in all the included studies, particularly in relation to pain and skeletal events. Parkes et al. (24) found that patients with multiple bone metastases experienced worse overall survival (OS) compared to those with a single bone metastasis (median OS = 4.80 years vs. 7.54 years, *p* < 0.0001). Moreover, patients with both axial and appendicular skeletal metastases had shorter OS than those with limited metastases confined to either the axial or appendicular skeleton (median OS = 4.58 years vs. 6.78 years, *p* < 0.0001). Berruti et al. (26) also incorporated the effects of bone metastases on survival, and found that pain levels and alkaline phosphatase were independent predictors of skeletal-related events (SREs). Cramer et al. (29) also uncovered that pain and the quality of life were poorer in patients who underwent trimodality treatment (surgery, radiation, and chemotherapy) and had depression and anxiety (OR of trimodality treatment = 3.55, *p* < 0.05; OR for depression = 3.91, *p* < 0.01; OR of anxiety = 4.22, *p* < 0.01).

### Pain assessment and measurement tools

Included studies used different pain measurement instruments to measure the intensity of pain and the effect of pain on patients. Numeric Rating Scales (NRS) were most frequently applied, with Tsai et al. (23) and Habib et al. (28) concluding that pain scores of 4 or higher were indicative of refractory cancer pain and worse outcomes. Meanwhile, Halabi et al. (27) utilized the Wisconsin Brief Pain Inventory (BPI) to measure pain interference, which was found to have a significant influence on OS. Furthermore, some studies included physician-assessed pain intensity and patients’ own ratings as the indicators of pain and pain-related interference. For instance, Parkes et al. (24) employed pain medication administration and clinician charting to measure pain at the time of bone metastasis diagnosis.

### Subgroup and multivariable analyses

The majority of the research conducted multivariate analysis to account for various factors, including age, sex, cancer type, and previous treatment. For instance, Tsai et al. (23) used multivariable logistic regression to determine independent predictors of clinical and radiographic response and showed that both Karnofsky performance status and lesion coverage volume were independent predictors. Parkes et al. (24) identified that the presence of multiple bone metastases and concurrent involvement of both axial and limb bones serve as risk factors for poor prognosis, correlating with significantly reduced overall survival rates. Furthermore, the research highlighted additional prognostic factors associated with adverse outcomes, including compromised systemic condition and elevated breast cancer grade, among others. Similarly, Cramer et al. (29) accounted for comorbid conditions and treatment modalities in their assessment of pain outcomes, emphasizing the critical role of patient characteristics in pain management.

## Data synthesis

### Incidence of refractory cancer pain

**Figure 2:**
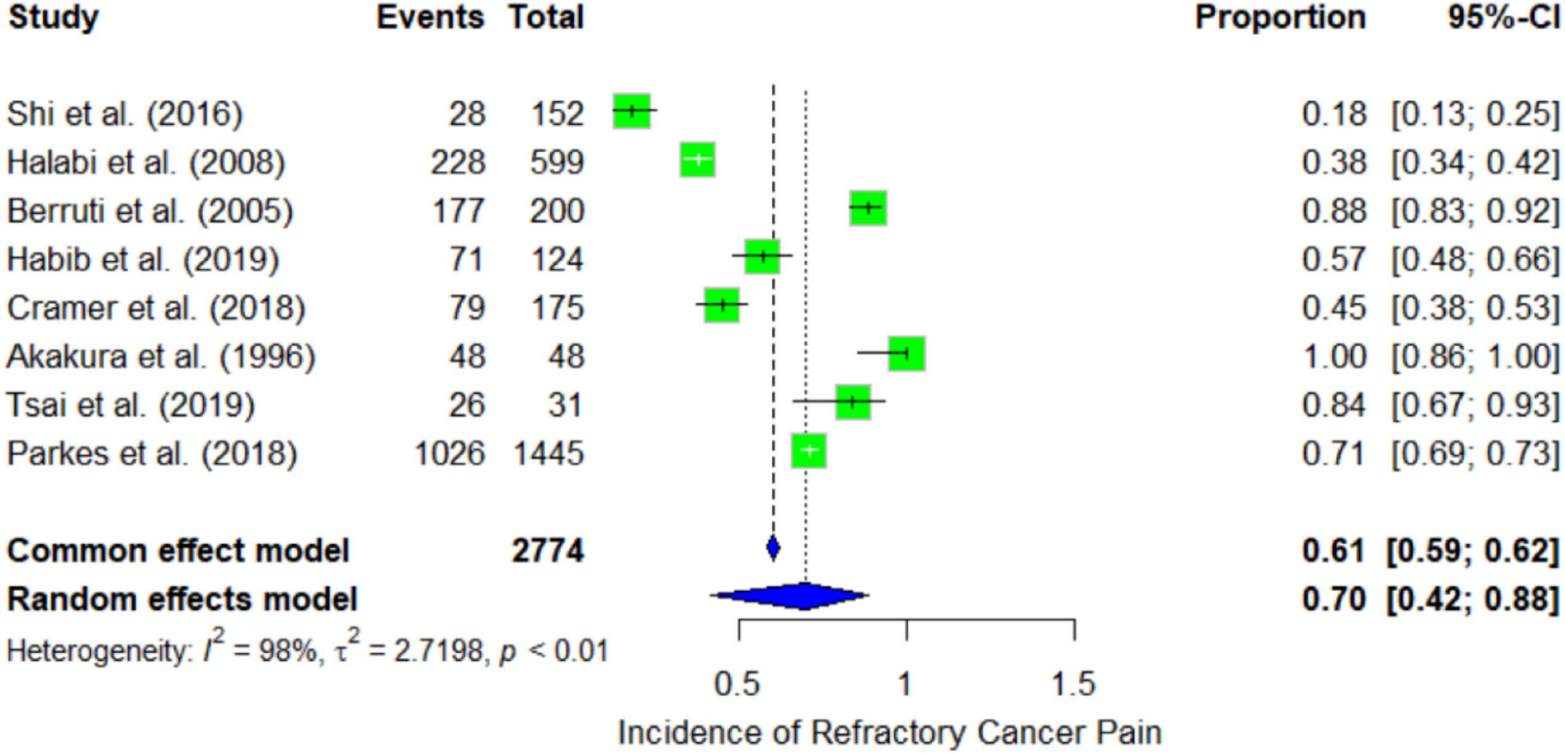
Forest plot representing the meta-analysis of the incidence of refractory cancer pain.

Our meta-analysis was conducted using a total of eight studies involving 2,774 patients. The individual incidence rates of refractory cancer pain were shown in Figure 2, Shi et al. (30) reported an individual incidence rate of 18.4%, while Akakura et al. (25) reported a rate of 100%. Utilizing a random-effects model, the pooled incidence was estimated to be 70%. The random-effects model produced a pooled incidence of 70%. This indicates that between 42 and 88% of patients with bone metastases continue to experience uncontrolled cancer pain despite standard management. The current meta-analysis demonstrated a high degree of heterogeneity, with *I*^2^ = 98%, indicating significant differences in the incidence of refractory cancer pain across the studies. The reason for high heterogeneity may be due to insufficient and missing data from grey research literature or unpublished studies. The *τ*^2^ value of 2.7198 further supports the presence of variability among the studies included in the analysis.

The common-effect model yielded a slightly lower pooled estimate of 61% (95% CI: 59 to 62%). The significant difference in results between the random effects and fixed effects models suggests substantial heterogeneity, indicating that the true incidence of refractory cancer pain may be influenced by study characteristics such as the cancer type, treatment modalities, and patient population.

**Figure 3:**
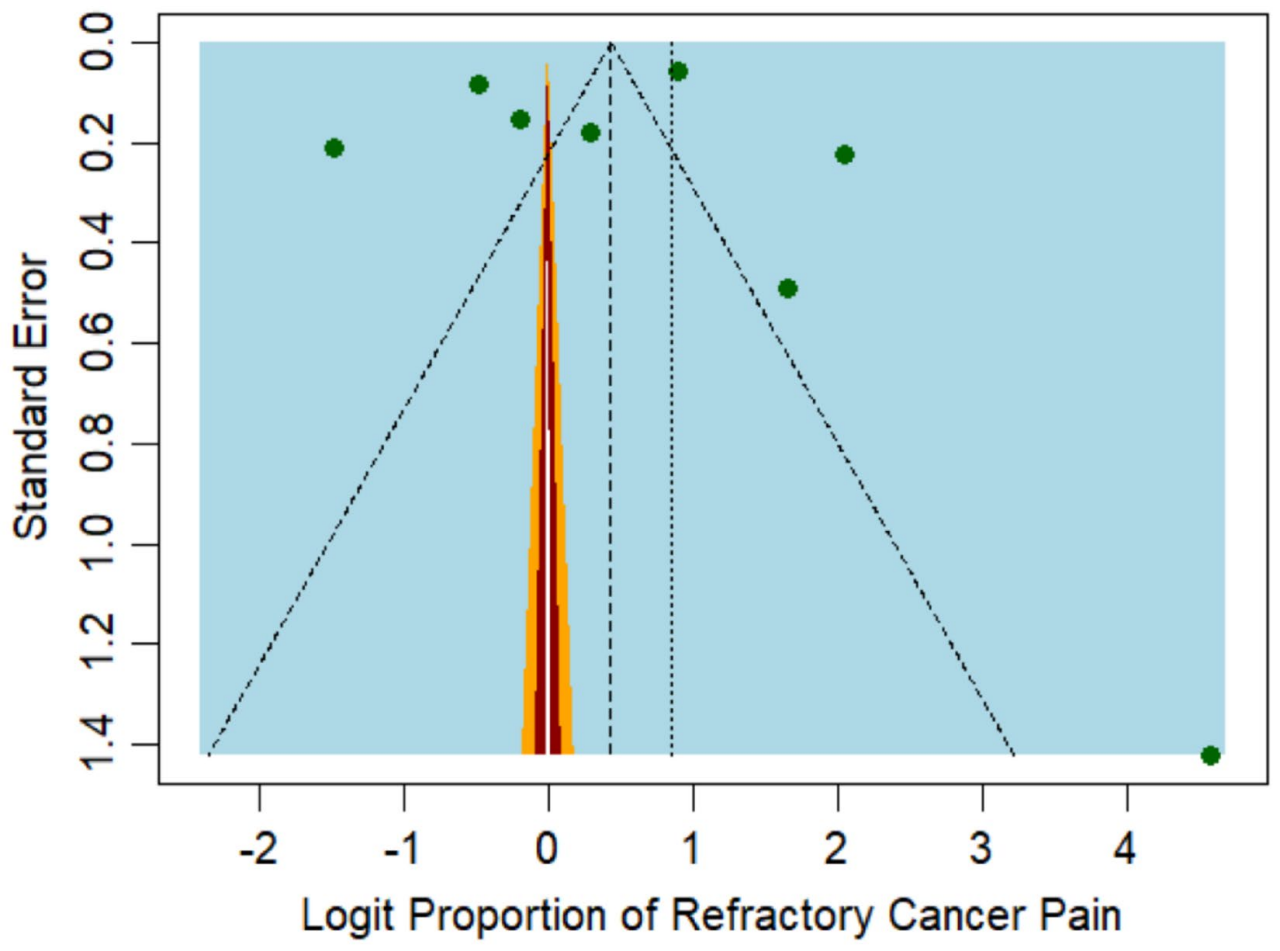
Funnel plot representing the publication bias among the studies reporting refractory pain incidence.

The funnel plot for incidence (Figure 3) exhibited some degree of asymmetry, potentially pointing to publication bias or other forms of small study effects. The primary analysis of our meta-analysis identified a higher incidence rate in studies with smaller sample sizes, such as the one by Akakura et al. (25), which reported an incidence rate as high as 91% among 48 patients. In contrast, studies with large sample sizes, such as Parkes et al. (24), which included 1,445 patients, reported more moderate incidence rates of 71%.

### Risk factors for refractory cancer pain

**Figure 4:**
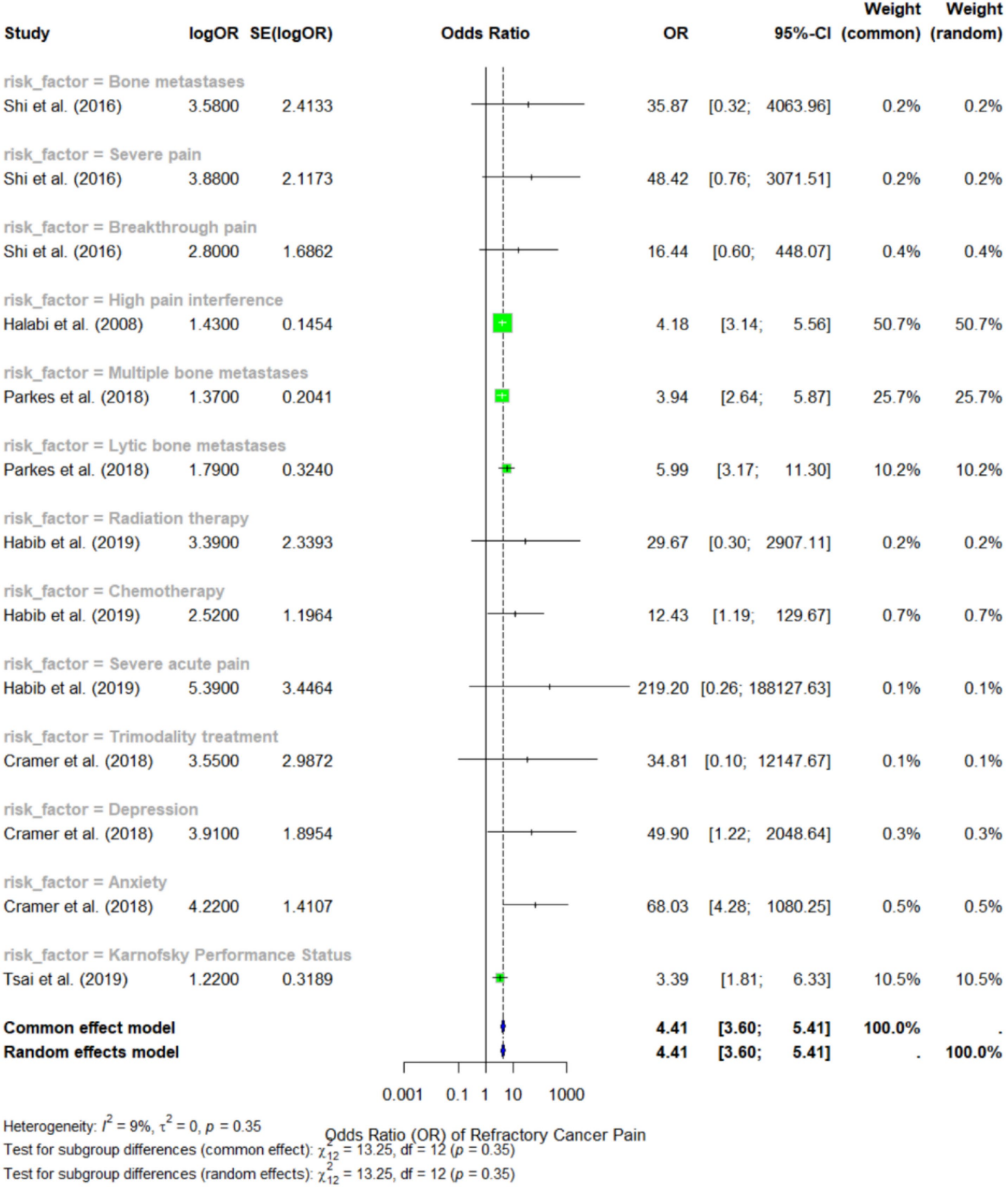
Forest plot representing the meta-analysis of the risk factors for refractory cancer pain.

In evaluating risk factors, the current cross-study meta-analysis identified 12 risk factors (Figure 4) across the included studies, and subgroup analyses were conducted for each factor. The random-effects model for the subgroup analysis yielded a pooled OR of 4.41 (95% CI: 3.60 to 5.41) for all the risk factors combined, indicating that the presence of these risk factors in patients with bone metastases increases the likelihood of developing refractory cancer pain.

Among the identified risk factors, severe acute pain showed the strongest association with refractory cancer pain, with an OR of 219.2 (95% CI: 0.26 to 188127.63), as reported by Habib et al. (28). However, the broad confidence interval indicates a high degree of variability in this estimate, likely due to small sample sizes and variability in measurement methods. Another notable predictor was breakthrough pain with an OR of 16.44 (95% CI: 0.60 to 448.07), according to Shi et al. (30), but this figure also demonstrated significant variation.

According to Parkes et al. (24), multiple bone metastases emerged as the most consistently reported risk factor across various studies, with an OR of 3.94 (95% CI: 2.64 to 5.87). The authors concluded that patients with extensive bone metastases are approximately 3.72 times more likely to experience refractory bone pain compared to those with limited bone lesions. Additionally, lytic bone metastases had a similarly strong association, with an OR of 5.99 (95% CI: 3.17 to 11.30), indicating that this type of bone lesion significantly increases the risk of refractory cancer pain.

**Figure 5:**
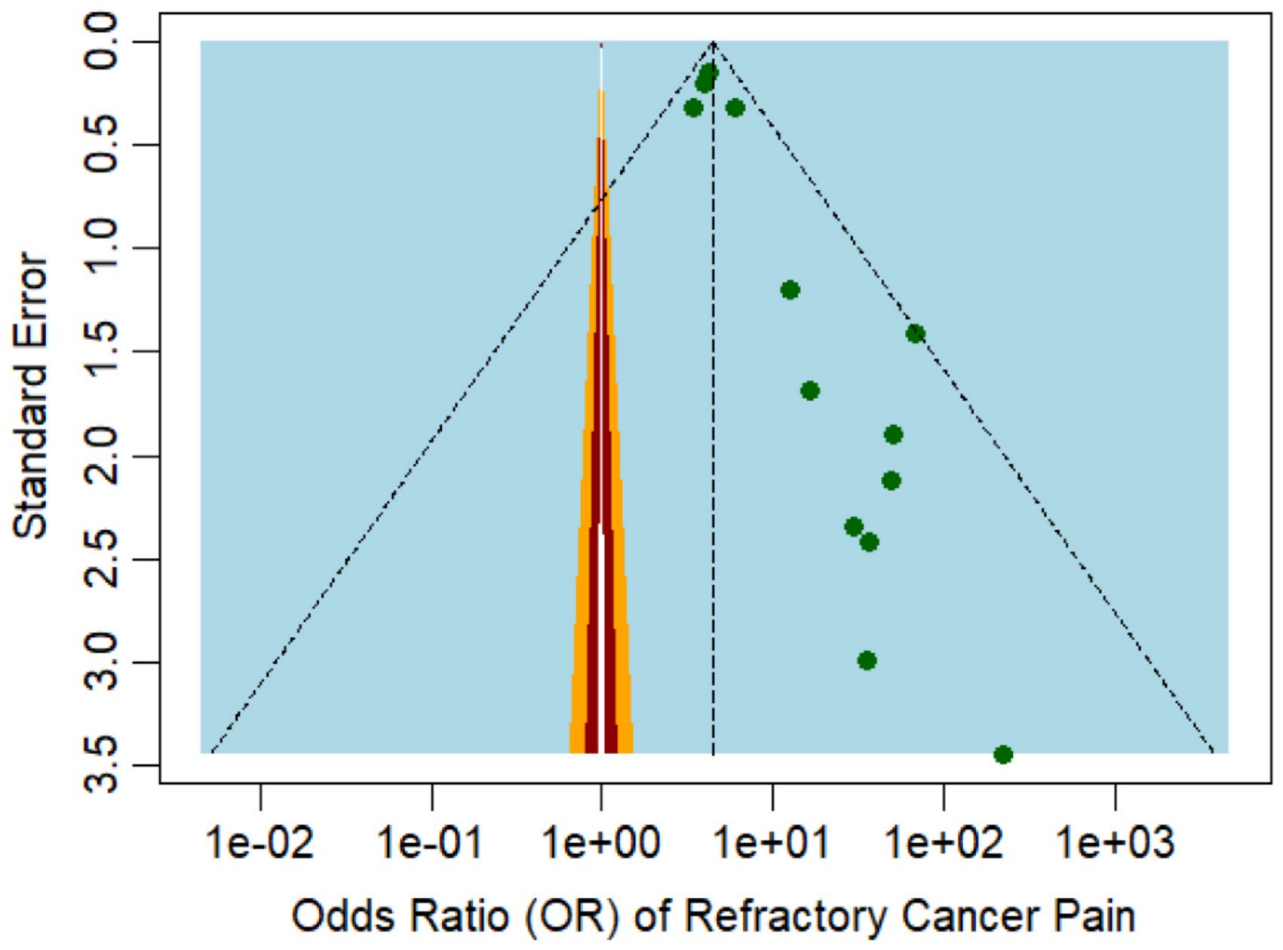
Funnel plot representing the publication bias among the studies that reported the risk factors of refractory cancer pain.

In terms of comorbidities, depression and anxiety were both identified as significant predictors of refractory cancer pain, with OR of 3.91 (95% CI: 1.22 to 2048.64) and 4.22 (95% CI: 1.22 to 2048.64), respectively, according to Cramer et al. (29). These findings suggest that pain evaluation should extend beyond physiological factors, as these may exacerbate pain perception and diminish the effectiveness of cancer treatment. The funnel plot in Figure 5 illustrates the study’s publication bias.

## Discussion

The present meta-analysis investigates the clinical factors influencing pain in patients with refractory bone metastases and their implications. By analyzing data from eight key studies encompassing various cancers and treatments, we identified significant risk factors affecting pain intensity, duration, and outcomes. Here, we will present the principal findings, compare them with existing literature, and explore their applications in pain management and patient care.

Our analysis identified key risk indicators for refractory cancer pain, including having more than two bone metastases, lytic bone lesions, severe acute pain, and psychological disorders such as depression and anxiety. Both multiple bone metastases and lytic lesions were independently associated with refractory cancer pain, underscoring the significant impact of skeletal involvement on pain severity and resistance in patients with metastatic cancer. Furthermore, given that overall outcomes are closely linked to psychological comorbidity rates, these findings emphasize the importance of addressing both physical and mental health issues in the pain management of cancer patients.

The incidence and risk factors for refractory cancer pain vary based on cancer type, treatment modalities, and patient characteristics, underscoring the necessity for personalized treatment plans for patients with bone metastases. Although most studies exhibited high methodological quality, there remain gaps in understanding refractory cancer pain and its optimal treatments. Future research should prioritize the identification of biopsychosocial interventions for cancer pain and explore non-opioid, unconventional pain management strategies. This meta-analysis emphasizes that refractory cancer pain represents a significant challenge for patients with bone metastases and identifies modifiable risk factors that can facilitate early detection and personalized management. Additionally, treatments for chemotherapy-induced peripheral neuropathy demonstrate improved outcomes when employing a multimodal approach that combines pharmacological and psychological methods.

### Key findings and interpretation

Among the studies included in this analysis, the authors identified the extent of bone metastases as one of the most significant determinants of pain. They found that patients with multiple bone metastases had a higher risk of experiencing refractory cancer pain compared to those with a single metastasis. For instance, Parkes et al. (24) showed that patients with multiple bone metastases were 1.37 times more likely to report pain than patients with single metastasis (95% CI; 1.02 to 1.84, *p* = 0.03). In a similar vein, Tsai et al. (23) identified lesion volume and Karnowski performance status as the most effective predictors of clinical and imaging response to treatment, thereby highlighting the significance of metastasis burden in relation to pain.

The results from these studies align with the existing knowledge that the extent and location of bone metastases are key determinants of pain and SREs. This is in tandem with the observation that multiple metastases are more destructive to bones as compared to single metastases, with the degree of destruction correlating with pain and other complications. Furthermore, metastases in both axial and appendicular skeleton, as described by Parkes et al. (24) have been shown to predict poor survival rates, highlighting the importance of comprehensive skeletal evaluation in clinical practice.

### Pain as a prognostic indicator

Another significant finding established in this meta-analysis is the prognostic value of pain in patients with bone metastases. Several studies have described the impact of pain intensity on survival, revealing that high pain intensity is associated with decreased survival. For instance, Halabi et al. (27) observed that patients with high pain interference had a significantly higher HR for mortality (HR = 1.43; *p* = 0.001), while Akakura et al. (25) found that time to requirement of analgesic was related with poorer survival (*p* < 0.01). This evidence suggests that pain not only reflects the burden of disease but may also serve as an indicator of disease progression that necessitates more intensive treatment in patients experiencing severe pain.

These findings are consistent with other research that has demonstrated a strong correlation between pain and survival in patients with metastatic cancer (11). Cancer-related bone pain is recognized as resulting from both local bone pathology and the overall tumor burden, as well as inflammation and immune response (31). Tumor-related pain encompasses both local and systemic factors, presenting challenges in the management of cancer pain and highlighting the need to improve patient outcomes.

### Impact of treatment modalities on pain management

The meta-analysis indicated that treatment modalities significantly influence pain-related outcomes. Based on randomized samples, studies focusing on patients undergoing aggressive treatments, such as chemotherapy and radiotherapy, demonstrated an increased likelihood of experiencing persistent or severe pain following these interventions. Habib et al. (28) observed a significant association between acute severe pain experienced during breast cancer surgery and the development of persistent pain (OR = 5.39, *p* = 0.001). Additionally, patients undergoing radiation therapy were found to be at an increased risk of long-term pain (OR = 3.39, *p* = 0.023). These findings underscore the importance for clinicians to incorporate pain management considerations into treatment planning, particularly for patients undergoing aggressive or multiple therapeutic interventions. Opioids were prescribed as the primary analgesic for patients experiencing severe pain, as documented by Halabi et al. (27) and Akakura et al. (25). Nonetheless, the reliance on opioids underscores the necessity for complementary or adjunctive therapies in the management of patients with challenging pain conditions. Pain management in patients with bone metastases often involves multimodal treatments, including the use of bisphosphonates and other bone-modifying agents. Notably, studies by Parkes et al. (24) and Tsai et al. (23) have demonstrated the efficacy of bisphosphonates and denosumab in preventing SREs and improving pain management.

### Predictors of persistent and severe pain

In the included studies, several variables were found to be significant risk indicators for patients with bone metastases experiencing persistent or severe pain. These included acute pain, the degree of bone involvement, performance status, comorbidities, and duration of pain. Notably, Cramer et al. (29) found that depression and anxiety were associated with more severe pain (OR of depression 3.91, *p* < 0.01; OR of anxiety = 4.22, *p* < 0.01). This finding underscores the importance of early intervention for psychological disorders that may heighten patients’ perception of pain prior to the initiation of cancer treatment.

In addition, we found that the type of bone metastases (lytic or blastic) could help predict the severity of pain experienced by patients. Parkes et al. (24) observed that patients with lytic metastases were 1.79 times more likely to report pain than patients with blastic metastases (95% CI 1.34–2.39, *p* = 0.001). Lytic lesions are known to be associated with more profound bone resorption, resulting in increased pain and decreased structural integrity of the skeletal system, hence the higher pain scores of the patients.

### Clinical implications for pain management

The findings of this meta-analysis yield several important clinical implications. First, identifying potential candidates for pain-related predictors in patients with bone metastases can assist clinicians in the initial evaluation and prevention of pain. Factors such as skeletal metastases, lytic lesions, and poor performance status should be utilized to identify patients at higher risk of developing pain; thus, patients exhibiting these characteristics should be considered for more intensive pain management strategies. Furthermore, the strong correlation between pain and survival underscores the necessity of effective pain management in patients with advanced cancer, as inadequate pain control may indicate disease progression and poor prognosis.

Bone-modifying agents like bisphosphonates and denosumab are used for pain relief and managing SREs (32). These agents are effective not only in preventing pathological fractures and spinal cord compression but also in controlling pain by stabilizing bone structure (33). Therefore, it is essential to incorporate these agents into standard management strategies for patients with bone metastases to enhance both the duration and quality of life.

Lastly, the appropriate management of psychological disorders, such as depression and anxiety, is crucial for individuals experiencing cancer pain. According to Cramer et al. (29), mental health issues significantly contribute to psychological distress, which can influence pain intensity. Addressing these disorders through various interventions such as counseling, medications, or cognitive behavioral therapy can enhance not only pain management but also the overall well-being of the patient.

### Limitations and future directions

Despite the valuable insights provided by this meta-analysis regarding the predictors of refractory cancer pain in patients with bone metastases, several limitations should be acknowledged. The included studies were different in terms of patient populations, cancer types, and treatment approaches, raising concerns about the generalizability of the results. Furthermore, most of the included studies were cross-sectional and predominantly descriptive in design, which may limit the ability to draw direct comparisons between risk factors and pain outcomes. Therefore, there is a need to conduct additional randomized controlled trials (RCTs) to establish pain management interventions for patients with bone metastases. Specifically, further research comparing the effectiveness of non-opioid treatments, including bisphosphonates, denosumab, and radiopharmaceuticals, on pain and survival is warranted. In addition, it is essential to thoroughly investigate the role of psychological and social factors in pain management, as these factors are crucial to understanding pain and patients’ overall experiences.

## Conclusion

This systematic review and meta-analysis aimed to identify and estimate the magnitude of risk factors for refractory cancer pain in patients with bone metastases, as well as to assess the prevalence of such pain across various studies. Our analysis revealed a pooled incidence of approximately 70% for refractory cancer pain, indicating that cancer pain remains a significant challenge even with conventional management approaches. This high prevalence underscores the urgent need for the development of improved and more personalized pain management strategies for this patient population.

## Data Availability

The original contributions presented in the study are included in the article/supplementary material, further inquiries can be directed to the corresponding author.
